# Genomic comparison of *Trypanosoma conorhini* and *Trypanosoma rangeli* to *Trypanosoma cruzi* strains of high and low virulence

**DOI:** 10.1186/s12864-018-5112-0

**Published:** 2018-10-24

**Authors:** Katie R Bradwell, Vishal N Koparde, Andrey V Matveyev, Myrna G Serrano, João M P Alves, Hardik Parikh, Bernice Huang, Vladimir Lee, Oneida Espinosa-Alvarez, Paola A Ortiz, André G Costa-Martins, Marta M G Teixeira, Gregory A Buck

**Affiliations:** 1 Center for the Study of Biological Complexity, Virginia Commonwealth University, Richmond, VA USA; 2 Present address: Institute for Genome Sciences, University of Maryland, Baltimore, MD USA; 3 Department of Microbiology and Immunology, Virginia Commonwealth University, Richmond, VA USA; 4 Department of Parasitology, ICB, University of São Paulo, São Paulo, SP Brazil

**Keywords:** Trypanosomatids, Comparative genomics, Genome sequencing

## Abstract

**Background:**

*Trypanosoma conorhini* and *Trypanosoma rangeli*, like *Trypanosoma cruzi,* are kinetoplastid protist parasites of mammals displaying divergent hosts, geographic ranges and lifestyles. Largely nonpathogenic *T. rangeli* and *T. conorhini* represent clades that are phylogenetically closely related to the *T. cruzi* and *T. cruzi*-like taxa and provide insights into the evolution of pathogenicity in those parasites. *T. rangeli*, like *T. cruzi* is endemic in many Latin American countries, whereas *T. conorhini* is tropicopolitan. *T. rangeli* and *T. conorhini* are exclusively extracellular, while *T. cruzi* has an intracellular stage in the mammalian host.

**Results:**

Here we provide the first comprehensive sequence analysis of *T. rangeli* AM80 and *T. conorhini* 025E, and provide a comparison of their genomes to those of *T. cruzi* G and *T. cruzi* CL, respectively members of *T. cruzi* lineages TcI and TcVI. We report de novo assembled genome sequences of the low-virulent *T. cruzi* G, *T. rangeli* AM80, and *T. conorhini* 025E ranging from ~ 21–25 Mbp, with ~ 10,000 to 13,000 genes, and for the highly virulent and hybrid *T. cruzi* CL we present a ~ 65 Mbp in-house assembled haplotyped genome with ~ 12,500 genes per haplotype. Single copy orthologs of the two *T. cruzi* strains exhibited ~ 97% amino acid identity, and ~ 78% identity to proteins of *T. rangeli* or *T. conorhini*. Proteins of the latter two organisms exhibited ~ 84% identity. *T. cruzi* CL exhibited the highest heterozygosity. *T. rangeli* and *T. conorhini* displayed greater metabolic capabilities for utilization of complex carbohydrates, and contained fewer retrotransposons and multigene family copies, i.e. trans-sialidases, mucins, DGF-1, and MASP, compared to *T. cruzi*.

**Conclusions:**

Our analyses of the *T. rangeli* and *T. conorhini* genomes closely reflected their phylogenetic proximity to the *T. cruzi* clade, and were largely consistent with their divergent life cycles. Our results provide a greater context for understanding the life cycles, host range expansion, immunity evasion, and pathogenesis of these trypanosomatids.

**Electronic supplementary material:**

The online version of this article (10.1186/s12864-018-5112-0) contains supplementary material, which is available to authorized users.

## Background

The class Kinetoplastea includes a broad spectrum of free-living and parasitic protists [1], all of which display unique features including trans-splicing, polycistronic transcription and RNA editing [2]. *Trypanosoma cruzi* is obligately parasitic, exhibits a broad mammalian host range, and is believed to have first infected and caused Chagas Disease in humans when the New World was populated ~ 15,000 years ago [3]. Usually spread by fecal contamination from an infected reduviid bug, the parasite replicates as intracellular amastigotes in a broad array of cell-types in its mammalian hosts [4]. It replicates as epimastigotes in the gut of its insect vectors, i.e. hemipterans of Triatominae such as species of the *Rhodnius*, *Triatoma* and *Panstrongylus* genera [5]. Clonal divergence [6, 7] and genetic exchange [8–10], have given rise to widely heterogeneous populations, termed Discrete Typing Units (DTU’s) TcI-TcVI and Tcbat (c.f. [11]). It is now generally believed that *T. cruzi* is a recent descendant of a phylogenetic lineage of closely related species of *Trypanosoma* tightly linked to bats [12, 13].

*T. conorhini* and *T. rangeli* are members of a phylogenetic group generally considered to be most closely related to the clade comprising *T. cruzi* and bat trypanosomes (*T. cruzi*-like) of the subgenus *Schizotrypanum*. Because of their phenotypes and the limited genetic information previously available, these two species of trypanosomes have been considered to occupy a phylogenetic position between the *T. cruzi*-like species and the African trypanosomes related to *T. brucei*. However, their close phylogenetic grouping is surprising given their strikingly different lifestyles. *T. conorhini* is spread to hosts in the feces of its vector the reduviid bug *Triatoma rubrofasciata* after replication in the insect gut [14, 15]. It is transmitted to a restricted host range in rats, where it causes a mild and transient infection, although it has also been reported to infect mice and non-human primates in the laboratory [15, 16]. The parasite and its vector are tropicopolitan, and there is a strong association of *Tr. rubrofasciata* with rats [14, 16]. In contrast to *T. cruzi* and like *T. conorhini*, *T. rangeli* exhibits an apparently exclusively extracellular lifestyle in its mammalian hosts. *T. rangeli*, like the African trypanosomes transmitted by tsetse flies, replicates as metacyclic trypomastigotes in the salivary glands of triatomine of the genus *Rhodnius* [17, 18], and is transmitted by a bite from an infected vector [19–21]. *T. rangeli* exhibits antigens in common with *T. cruzi*, and likewise is widely distributed in Central and South America with a broad mammalian host range that includes humans [18]. Five phylogenetic lineages of *T. rangeli* have been identified; TrA, C, D and E are phylogenetically close, but TrB (which includes the AM80 strain reported herein) is a more divergent lineage positioned basal to the clade formed by all lineages of *T. rangeli* [22–27]. *T. rangeli* isolates and local vectors have apparently co-evolved [18, 23, 24], with consequent parasite lineage association with *Rhodnius* complexes [18, 23–25, 28]. Infection of mammalian hosts by *T. rangeli* is non-pathogenic and induces low parasitaemia, but can persist for years [29]. Mammalian host-parasite interaction mechanisms remain largely unclear for both *T. conorhini* and *T. rangeli*.

Because of their taxonomic positions and diverse lifestyles, these parasites present an opportunity to identify the genetic bases of their differing abilities to invade cells, evade host immune responses, and cause disease, and their diverse host ranges and life cycles in mammals and vectors. Comparisons of dixenous trypanosomatids to free-living bodonids have suggested that most differences lie within genes encoding metabolic and surface proteins [30]. Genome analysis of *Leishmania major* Friedlin, *Trypanosoma brucei* TREU 927 and *T. cruzi* CL Brener [31–34], studies of lineage-specific features in *T. cruzi* Sylvio X10/1 (TcI) and *T. cruzi* CL Brener (TcVI) [35], and comparisons of *T. cruzi* and the bat-restricted *T. cruzi marinkellei* [36] suggest many differences are associated with differential multigene family expansion. More recently, a sequence draft of *T. rangeli* SC-58, a representative of the TrD lineage isolated from rodents and never found in humans [37], was presented [38]. The *T. rangeli* AM80 strain was isolated from a human source in the Amazon [39], where the TrB lineage, the basal and most divergent of all known *T. rangeli* lineages, is highly prevalent. Lineages TrA, prevalent from the northwestern region of South America (including Brazilian Amazonia) to Central America, and TrC spanning from the west of the Andes to Central America, have also been found in humans. Lineages TrD and TrE were rarely reported and so far only isolated from wild mammals and triatomines [22–24, 28, 40, 41]. Study of a TrB strain, e.g. AM80, would likely present one of the most topical and timely comparisons to other trypanosomatid groups in terms of relevance to human infection. Moreover, the ongoing rapid development of the Brazilian Amazon is likely to impact transmission of both *T. rangeli* and *T. cruzi* to humans, which are commonly co-infected with both parasites [23, 26, 40].

In this work, we sequence and compare the genomes of *T. rangeli* AM80, *T. conorhini* 025E, *T. cruzi* G (TcI) and *T. cruzi* CL (TcVI, a clone from the same parental strain as the published CL Brener strain [32]) (see Additional file 1: Table S1 for strain information). These two *T. cruzi* isolates present a disparate range of characteristics: *T. cruzi* G isolated from a marsupial, displays low parasitemia in vivo [42] and induces chronic infection of low virulence in mice [42], exhibits higher susceptibility to interferon-γ [43], and has a lower ratio of cruzipain to chagasin [44]. *T. cruzi* CL was derived from a triatomine bug captured in the residence of a chagasic person [45], is likely a hybrid of isolates from TcII/III lineages [9, 10, 46–48], and exhibits high parasitemias and virulence. *T. cruzi* G uses mucin-like glycoproteins to facilitate cell invasion, while the CL strain uses the stage-specific gp82 surface molecule and cruzipain [49, 50].

Our analyses of the sequences of these four parasites suggest disparate assembled genome sizes ranging from ~ 21–65 Mbp and extend previous observations that *T. cruzi* CL is a hybrid strain [11, 32, 48]. In contrast to many *T. cruzi* strains, we found no evidence of hybridization in the genomes of *T. rangeli* and *T. conorhini*. By comparing the genomes of these three species we aimed to infer how these genomes have evolved since their last common ancestor and to gain insight into the selective pressures acting upon them. Our data have led us to consider the hypothesis that higher levels of heterozygosity in protein-coding genes of *T. cruzi* CL impart an adaptive advantage. We further hypothesize that lower diversity in multigene families, and gene clusters defined by sequence similarity, may help explain the more restricted host range of *T. conorhini* 025E. Our results lay the groundwork for further studies to elucidate the genetic basis for the phenotypic differences among these closely related kinetoplastid taxa.

## Results and discussion

### Genome assemblies and molecular karyotypes

#### Molecular karyotypes

**Fig. 1:**
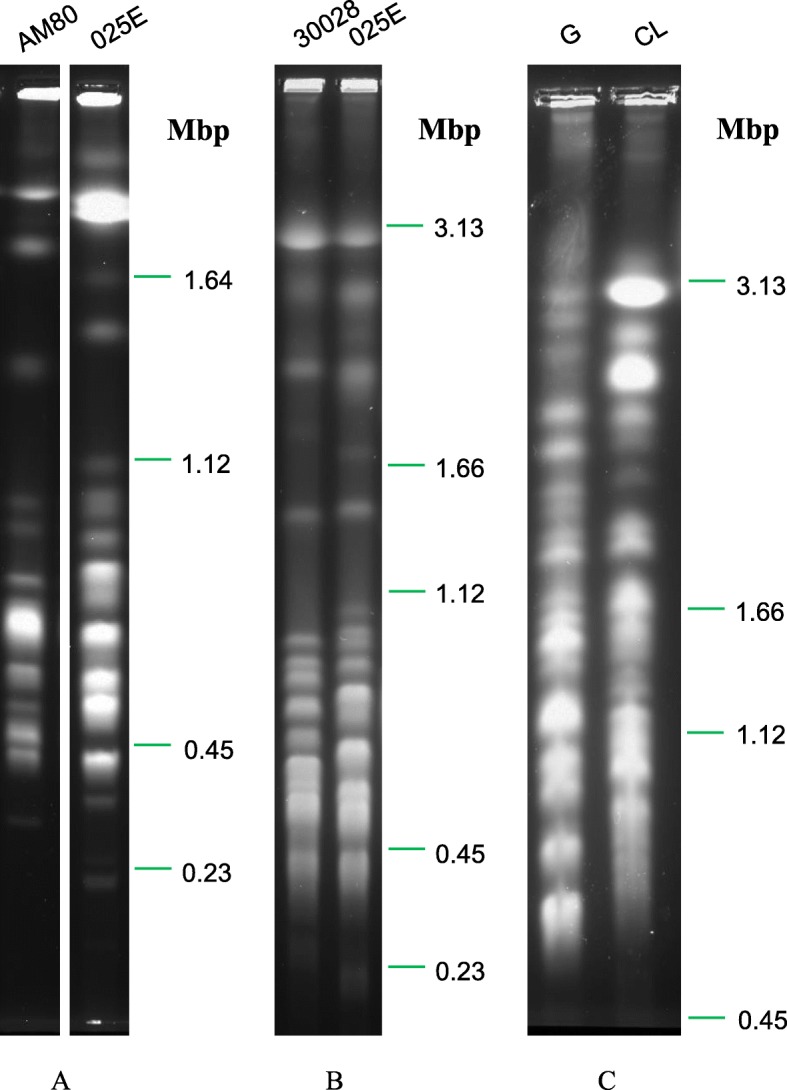
Karyotypes from three PFGE runs. 1% Megabase agarose gels (Bio-Rad) were loaded with agarose plugs bearing lysates of ~ 1 × 10^7^ epimastigotes of each trypanosomatid strain for electrophoresis at 13.5 °C using the CHEF DR III System (Bio-Rad). Run conditions used for karyotyping each species were based empirically on their individual distributions of chromosome sizes. For separation of smaller chromosome size ranges, we used the following program - Block 1: 5 V/cm, 20–200 s, 18 h, 120°. Block 2: 3 V/cm, 200–300 s, 32 h, 120°. Block 3: 1.5 V/cm 500–1100 s, 12 h, 120°. The program used for separation of the largest chromosome size ranges was as follows - Block 1: 2 V/cm, 1500 s, 12 h, 98°. Block 2: 2 V/cm, 1800 s, 12 h, 106°. Block 3: 3 V/cm, 500 s, 38 h, 106°. Block 4: 5 V/cm, 20–200 s, 23 h, 120°. Block 5: 3 V/cm, 200–400 s, 34 h, 120°. (**a**) *T. rangeli* AM80 vs. *T. conorhini* 025E using *Saccharomyces cerevisiae* chromosome size-markers (Bio-Rad). (**b**) *T. conorhini* 30028 vs. *T. conorhini* 025E. (**c**) *T. cruzi* G vs. *T. cruzi* CL. *Schizosaccharomyces pombe, Hansenula wingei* and *Saccharomyces cerevisiae* chromosomes (Bio-Rad) were used as markers for (**b**) and (**c**)

Pulsed Field Gel Electrophoresis (PFGE) under multiple conditions provided an estimate of the sizes and numbers of chromosomes in the genomes of *T. conorhini* 025E, *T. rangeli* AM80, *T. cruzi* G, *T. cruzi* CL, and an additional isolate, *T. conorhini* 30028, obtained from ATCC (Fig. 1, Table 1, and Additional file 1: Table S2). *T. conorhini* ATCC 30028 was isolated in Hawaii in 1947 from *Triatoma rubrofasciata*. Pixel intensity and area under the curve (“volume”) of each band were plotted against the distance migrated in the gel and compared to standard curves of presumed single-copy diploid chromosome band volumes to provide an estimate of the copy number of each chromosome. Bands with estimated areas that were half of that expected for a chromosome pair were found in all species and were assumed to be due to size differences in chromosome pairs, as has previously been observed in *T. cruzi* [51–55]. NGS estimates for *T. cruzi* genome size are given separately for its two haplotypes (Esmeraldo-like and Non-Esmeraldo-like).

**Table 1:** PFGE and densitometry summary

	*T. conorhini* 025E	*T. conorhini* 30028	*T. rangeli* AM80	*T. cruzi* G	*T. cruzi* CL
Number of bands	21	20	19	18	21
Number of predicted chromosomes^a^	45	39.5	40	36.5	37
Number of chromosomes >1 Mbp	10	8	7	19.5	30
Number of chromosomes 300 Kbp to 1 Mbp	33.5	31.5	33	17	7
Number of chromosomes <300 Kbp	1.5	0	0	0	0
Size range of chromosomes (Mbp)	3.22–0.21 (3.01)	3.24–0.33 (2.91)	3.24–0.31 (2.92)	3.09–0.66 (2.43)	3.48–0.74 (2.75)
Predicted genome size (Mbp)^b^	39.70	38.42	34.85	44.01	61.48
NGS estimate for genome size (Mbp)^c^	41.04	n/a	30.33	48.75	41.81 (ESM-like), 44.74 (NonESM), 55.51 (Unassigned)

^a^Sum of the copy numbers predicted for each band by densitometry (see Methods)

^b^Sum of the total predicted number of Mbp at each band

^c^(Total number of bases) / (modal alignment depth of the assembly)

These analyses suggest that whereas *T. cruzi* CL and G each bear ~ 37 chromosomes, similar to that previously estimated for *T. cruzi* CL Brener [52]. *T. rangeli* AM80, *T. conorhini* 30028, and *T. conorhini* 025E have a slightly greater number; i.e. 40, 39.5 and 45 chromosomes, respectively. Although the chromosome numbers of the G and CL strains are quite conserved, the sizes of the individual chromosomes are not, following a trend previously predicted for *T. cruzi* strains [52]. Previous studies have revealed significant variation in PFGE patterns specific to distinct lineages of *T. rangeli* [26, 56]. Genome sizes determined as described (Table 1, Additional file 1: Table S2) are estimates, but closely match estimates from sequence analysis.

As expected, significant genome variability was observed among these karyotypes, although the two *T. conorhini* isolates show similar banding patterns (Fig. 1). Clearly, major chromosomal rearrangements, expansions, or deletions seem to have occurred during the evolution of these parasites. Interestingly, the two *T. cruzi* strains appear to have at least double the number of megabase-sized chromosomes as *T. conorhini* or *T. rangeli*, and although the latter parasites have more chromosomes than the *T. cruzi* strains, their overall genome sizes are reduced. Repeat expansions in individual chromosomes have previously been invoked to describe karyotype polymorphism across *T. cruzi* strains [53, 55, 57]. Despite these differences we found single copy ortholog genes to be highly syntenic in these genomes (data not shown).

### Genome characteristics

Each of these genomes was sequenced as described in the Methods and analyzed for completion and integrity using an in-house genome completion assessment pipeline called GenoCIA, which demonstrated that all or nearly all of the genes from each of these organisms are represented full-length and intact in our assemblies (Additional file 2: Figure S1). The results are summarized in Table 2 and Additional file 1: Table S3.

**Table 2:** Principal genome characteristics for *T. conorhini* 025E, *T. rangeli* AM80*, T. cruzi* G and *T. cruzi* CL

	*T. conorhini* 025E	*T. rangeli* AM80	*T. cruzi* G	*T. cruzi* CL ESM-like	*T. cruzi* CL NonESM	*T. cruzi* CL Unassigned
Sum of # bases in all contigs (Mbp)^a^	21.34	21.16	25.18	26.77	27.98	10.26
GC Content (%)	57.24	51.96	50.06	50.31	50.44	53.45
Coding Region (Mbp)	14.25	13.61	16.23	14	14.77	5.85
Coding Region (%)	66.78	64.32	64.46	52.30	52.79	57.02
Number of Protein-coding Genes	10,154	10,109	12,712	12,229	13,066	6993
Orthologous groups^b^	9055	9140	10,103	19,790		
Total number of contigs	1660	1080	1452	2387	2290	3087
N50 length	24,561	43,151	74,655	73,547	83,750	8012
N50 No. contigs	257	157	91	95	95	294
N50 avg. contig length	41,520	67,443	138,662	141,304	147,390	17,455
Genes w/ Pfam hits	8610	7187	8055	7425	7734	3744

^a^Repetitive and complex regions may not be uniquely assembled

^b^Gene Clusters (orthologs, paralogs, singletons) derived from an OrthoFinder run using all four species

The genome assembly sizes of these organisms range from ~ 21 Mbp for *T. conorhini* and *T. rangeli* to 25–65 Mbp for *T. cruzi* G and CL, respectively. Discrepancies between the assembly size in Table 2 and the estimated genome size from Table 1 were observed. As previously reported for the genome of *T. cruzi* CL Brener [32], this discrepancy has been ascribed to the collapse of near-identical repeats into fewer copies in the assembly. The genomes reported herein likewise contain such highly repetitive sequence. The genomes of *T. cruzi* G, *T. conorhini*, *T. cruzi* CL Esmeraldo-like and *T. cruzi* CL Non-Esmeraldo-like each exhibited collapsed repeats, with ~ 20–30% of all bases in their assemblies exhibiting > 1.5X, 5–15% >3X, and 0.5–0.8% > 10X coverage relative to the average coverage of their single copy orthologs. *T. rangeli*, which had the least discrepancy between assembly size and estimated genome size, had values of 12% > 1.5X, 4% >3X and 0.6% >10X the coverage of its single copy orthologs, and for *T. cruzi* CL Unassigned, which had the most discrepancy between the two sizes, these values were significantly higher, at 44%, 27% and 4%, respectively. Thus, we assume that these repetitive sequences largely explain the discrepancy between the genome assembly size and the estimated genome size from PFGE.

The GC content of *T. conorhini* was slightly higher at ~ 57% than for the other three genomes, which ranged from ~ 50–52%. The number of genes in *T. conorhini* and *T. rangeli* (~ 10,000) is less than the number in *T. cruzi* G or CL (13,000), and is largely consistent with that observed in other kinetoplastid protozoa [31–33] (see Additional file 1: Tables S4−7 for all genes and their annotation). *T. cruzi*, however, is recognized for having many large multigene families [32, 35] likely explaining the expanded repertoire in the G and CL strains. Additionally, the CL strain is considered a hybrid [48], with a larger genome size than typically found in TcI strains [57, 58], consistent with our observations of genome size and gene content.

The genomes of each of these organisms apparently contain genes required for meiosis, suggesting likely capacity for sexual reproduction (Additional file 1: Table S3). Counts of glycosylphosphatidylinositol (GPI) anchored proteins and proteins with transmembrane domains are very similar between *T. conorhini* 025E and *T. rangeli* AM80, and highest in the *T. cruzi* strains. 18S rRNA copy numbers show excellent agreement between bioinformatics estimates and measurements by quantitative PCR. We estimated that there are 4–7 copies in *T. cruzi* G and 2–3 copies in *T. cruzi* CL. The *T. cruzi* CL Brener genome assembly contains 12 fragments of 18S rRNA genes in TriTrypDB [59] v.28, all less than half the size of the predicted 18S genes from the CL and G strains (~ 2300 bp). Estimates based on sequence alignment to a 186 bp highly conserved region of the spliced leader gene transcript, suggested that the genomes of *T. cruzi* G, *T. cruzi* CL, *T. rangeli* AM80, and *T. conorhini* 025E have 66, 82, 44 and 13 copies of the spliced leader gene, respectively.

### Sequence identity and phylogenetic analysis

**Fig. 2:**
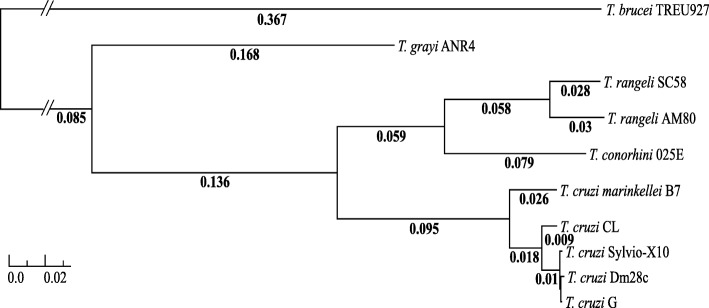
Maximum Likelihood tree from 139 aligned and concatenated amino acid sequences. Support values are calculated from 1000 bootstrap replicates, all bootstrap values were 100% and thus not displayed. Scale bar indicates mean number of substitutions per site. A break in the branch to *T. brucei* was used to aid visualization since the branch was too long to display

The relationships among these parasites and their phylogeny were explored using 139 single copy orthologs identified as outlined in the Methods from each of these parasites and six closely related trypanosomatids (Fig. 2, Additional file 1: Tables S8 and S9). Our analyses showed that *T. conorhini* 025E and *T. rangeli* exhibited ~ 84% identity to each other, and only 77% identity to *T. cruzi* isolates. Percent nucleotide identity between the TraB (AM80) and TrD (SC58) isolates of *T. rangeli* was 92%. As expected, the highest identities observed, i.e.*,* 94–98%, were between the *T. cruzi* isolates, with two DTU I isolates, G and Sylvio, being the most similar, aside from *T. cruzi* strains CL and CL Brener, which are clones from the same strain and exhibit near 100% identity (not shown). Our observations support previous reports of *T. brucei* and *T. cruzi marinkellei* B7 percent identities to strains of *T. cruzi* [34, 36]. Interestingly, the ~ 92% percent identity between *T. rangeli* AM80 and *T. rangeli* SC-58 was similar to the results comparing *T. c. marinkellei* and the *T. cruzi* strains.

Phylogenetic analysis (see Methods, and Fig. 2) using amino acid sequences of these 139 orthologs confirmed previous phylogenetic analyses based on a few genes [12, 13, 26–28], showing a close relationship between *T. rangeli* and *T. conorhini*, and a greater evolutionary distance between them and *T. brucei* clades than the distance between the *T. cruzi* and *T. brucei* clades. As expected, the *T. cruzi* strains are the most closely related, with the closest relationship between the two DTU I isolates, G and Sylvio. The subspecies *T. cruzi marinkellei* is clearly divergent from the *T. cruzi* strains. Given the relatively higher number of genomes available within the *T. cruzi* clade, a greater taxon sampling of genomes closely related to *T. conorhini*, *T. rangeli* and species more closely related to the *T. brucei* clade, which are not yet available, would have provided a more accurate and complete phylogenetic reconstruction.

### Gene cluster diversity

**Fig. 3:**
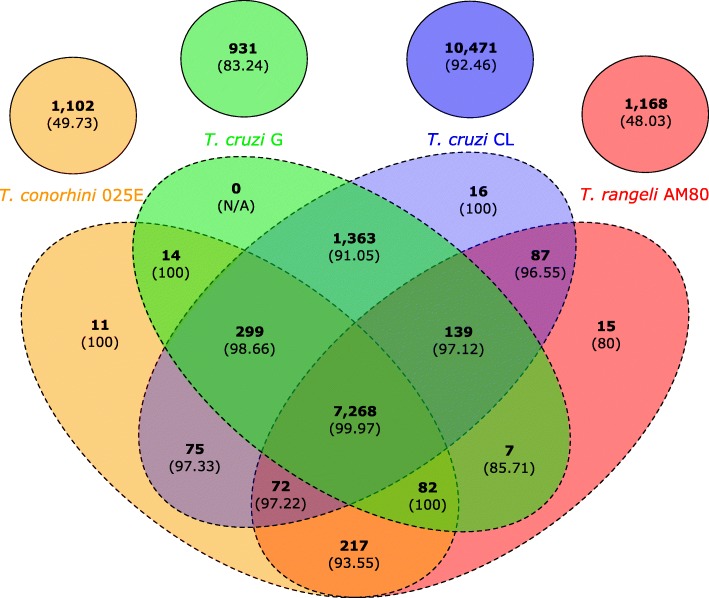
Sequence diversity and functional enrichment across clustered genes. Called genes from each species were clustered using OrthoFinder v.0.7.1. This yielded 23,337 clusters, shown here using draw.quad.venn from the VennDiagram package of Rstudio v.3.0.2, together with percent hits to TriTrypDB v.24, KOG or PFAM databases in parentheses. Circles with solid lines indicate singleton genes (no paralogs or orthologs, i.e. classified as a ‘cluster of one’ for this analysis), ovals with dashed lines represent clusters of genes that have orthologs and paralogs

Our OrthoFinder analysis suggested that the majority of orthogroups are represented by single genes for each organism present (i.e. absence of paralogs), although the number of clusters containing two paralogs is higher in *T. cruzi* CL (Additional file 2: Figure S2). Interestingly, the percentage of clusters containing genes annotated as surface proteins was generally proportional to the number of predicted copies in the cluster (Additional file 2: Figure S2), consistent with previous observations that surface protein genes exposed to immune surveillance are often highly repetitive [32, 38, 60–62]. Examining the species represented in each gene cluster (Fig. 3) identified 7268 gene clusters common to all four of the genomes. In contrast, there were 11,418 clusters unique to either the G or CL strains of *T. cruzi,* and 12,781 gene clusters unique to *T. cruzi*. *T. cruzi* CL alone exhibited 10,487 unique clusters, probably due to its hybrid genome. *T. rangeli* AM80 shares a total of 7655 clusters with the two *T. cruzi* strains, and *T. conorhini* 025E shares 7810. These results are suggestive that the genomes of *T. rangeli* and *T. conorhini* have undergone less gene amplification and divergence than has been reported for *T. cruzi* strains [34].

The most common BLASTp hits for the *T. rangeli* AM80 paralog-containing clusters include hypothetical proteins and the retrotransposon hotspot protein (RHS). For *T. rangeli* AM80 singletons, the most frequent possible homologs in NCBI’s non-redundant (nr) protein database are hypothetical proteins (208 clusters), trans-sialidase or *T. rangeli* sialidase (24 clusters), gp63 (20 clusters), and adenylate cyclase (10 clusters). The latter is important in the differentiation process of *T. cruzi* [63].

Around 20% of the 10,487 *T. cruzi* CL strain-specific gene clusters are from trans-sialidase, RHS, dispersed gene family 1 (DGF-1), mucin-associated surface protein (MASP), mucin and gp63 multigene families. It is interesting to note in *T. rangeli* AM80 the complete absence of BLAST hits <1e-5 for MASP and DGF-1 for singletons and clusters containing paralogs, and much fewer mucin and RHS hits than for *T. cruzi* CL. This result implies that in *T. rangeli* AM80 the MASP and DGF-1 genes have been lost or have diverged significantly. *T. cruzi* G, as for *T. cruzi* CL, contains many clusters with hits to trans-sialidase and RHS family members. The presence of many surface protein genes in the species-specific categories of *T. rangeli* AM80 and *T. cruzi* potentially contributes to their wide host range and ability to sustain infection in the mammalian host.

### Multigene family copy number

**Fig. 4:**
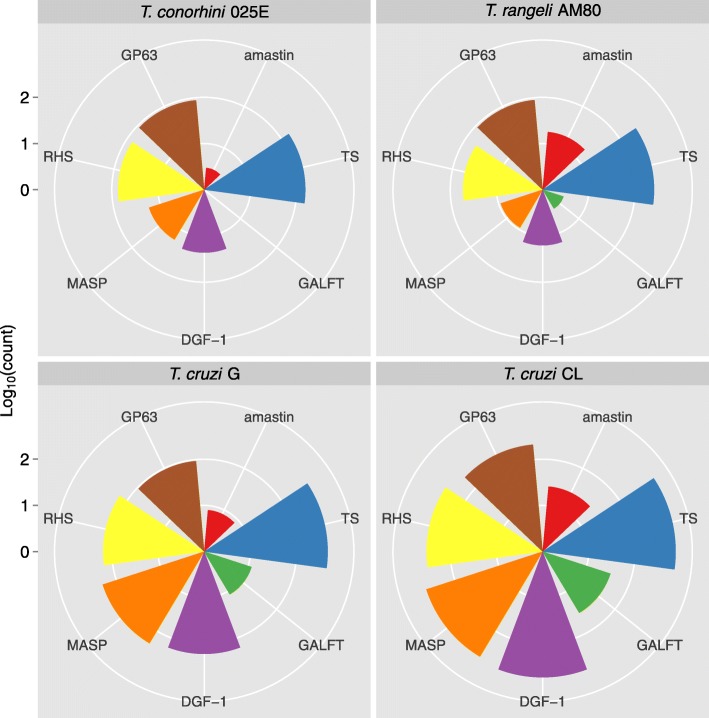
Multigene family copy numbers. Selected major multigene families shown are amastin, β-galactofuranosyl transferase (GALFT), surface protease GP63, retrotransposon hot spot (RHS) protein, mucin-associated surface protein (MASP), trans-sialidase (TS), and dispersed gene family protein 1 (DGF-1). Centers of plots represent 1 copy (0 in log_10_) and successive concentric circle values are shown by the log_10_ scale bar on the left

Like previous reports about *T. cruzi* [35, 36, 64], both *T. rangeli* AM80 and *T. conorhini* have variable representations of genes in multigene families (Fig. 4 and Additional file 1: Table S10). We found trans-sialidase (TS) and GP63 genes are highly expanded in all genomes we examined herein. The TS family genes, which encode proteins that are linked to the cell membrane via GPI anchors, are very heterogeneous and form eight known groups [65–67]. The enzyme in *T. cruzi* transfers host sialic acids to parasite cell surface ligands, presenting a decoy to the host immune response and participating in the adhesion and internalization of the parasites into host cells [61, 68–70]. *T. rangeli* has a Group II sialidase that is a strict hydrolase lacking the ability to transfer sialic acid [38, 65, 71–73]. In both species, TS Group II enzymes likely participate in host cell adhesion and invasion, but for *T. rangeli* this activity is probably not required in the mammalian host, but may be relevant in the triatomine vector [71, 73]. The sequences of *T. conorhini* TS were the most divergent compared to those from *T. cruzi, T. cruzi-*like species and *T. rangeli* [65]. The findings from this and previous studies uncovering TS genes in all *Trypanosoma* species, many of which do not invade mammalian cells, suggest that, in addition to participation in host cell invasion and intracellular survival, TS may play other roles in parasite development, e.g. in their arthropod vectors [64]. GP63 proteins are zinc-dependent metalloproteases that are highly expressed in *T. cruzi* amastigotes, where they contribute to cell infection [62, 74]. This activity is consistent with our observation that pathogenic *T. cruzi* CL has the highest number of copies of this gene (~ 211), similar to the 174 copies predicted in *T. cruzi* CL Brener [32, 75]. However, the role(s) of GP63 in *T. conorhini* and *T. rangeli* are unknown.

The most striking differences in copy number across the species are arguably in the mucin-associated surface protein (MASP) and dispersed gene family 1 (DGF-1) families, which are significantly less amplified in *T. rangeli* and *T. conorhini* than in the *T. cruzi* strains. In *T. cruzi*, MASP genes are often found in clusters with mucin and other surface protein genes [32], and the protein is localized to the surface of infective forms of the parasite [60]. Polymorphism of MASP amino acid sequence is high, which likely contributes to immune system evasion [60] and the ability to infect multiple cell types [64, 76, 77]. Thus, the smaller size of the MASP gene family in *T. conorhini* 025E and *T. rangeli* AM80 (and *T. rangeli* SC-58 [38]) may be related to their lack of host cell infectivity, and their inability to induce acute infections with high levels of parasitemia or long chronic infections. DGF-1 is less well represented in *T. rangeli* AM80 than in the other species, and shows lower diversity in gene cluster analysis in this study. Possession of only 16 copies of DGF-1 may contribute to the obligate extracellular nature of *T. rangeli* AM80 in the mammalian host, since this protein has been implicated in the ability of parasites to bind to extracellular matrix proteins of host cells [78]. *T. rangeli* SC-58 was previously estimated to have over 400 copies of this gene, despite less than 20 partial DGF-1 genes being annotated with genome coordinates [38]. If the latter copy number estimate is accurate, there is a striking inter-strain difference. *T. conorhini*, bearing only 23 copies, also shows significantly reduced numbers of DGF-1 compared to *T. cruzi*, which is likewise consistent with an extracellular lifestyle.

Cruzipain, a key player in cell invasion, and GALFT, which is involved in GPI anchor biosynthesis, are also highly differentially expanded. We find no evidence of cruzipain expansion in *T. rangeli* AM80 or *T. conorhini* 025E, although cruzipain homologs are present in these genomes. In *T. rangeli*, the homolog is known as rangelipain and is present in tandem repeats [26]. Amino acid identities for these genes are 76% between *T. rangeli* AM80 and *T. conorhini* 025E, 71% between *T. conorhini* 025E and the *T. cruzi* strains, and 69% between *T. rangeli* AM80 and the *T. cruzi* strains. We previously inferred network genealogies showing that cruzipain sequences of all DTUs of *T. cruzi* clustered tightly together and closer to *T. c. marinkellei* than to *T. dionisii* (*T. cruzi*-like species), but differed from homologs of *T. rangeli* and *T. brucei*, revealing DTU- and species-specific polymorphisms [79]. Cruzipain precursors are activated upon removal of the N-terminal prodomain, resulting in proteins linked to the invasion process that are thought to play a larger role in *T. cruzi* CL, where expression levels are higher during infection, than *T. cruzi* G [44]. However, we do not see a notable expansion of cruzipain precursors in the CL strain, which has ~ 50 copies, compared to ~ 38 copies in *T. cruzi* G.

We find a lower copy number of amastin in *T. conorhini* 025E (~ 3 copies) compared to *T. rangeli* AM80 (~ 18 copies), *T. cruzi* G and CL (~ 8–26 copies), and *T. cruzi* CL Brener (14 copies) [80]. Although the exact function of amastins remains unclear, they are thought to be abundantly expressed on the surface of intracellular *T. cruzi* amastigotes and apparently support intracellular survival [64, 81–85]. Since amastin is expressed in the intracellular mammalian amastigote stage of the parasite’s life cycles in *T. cruzi* and *Leishmania*, finding expansion of this immunogenic gene family in the extracellular *T. rangeli* AM80 (also previously reported in *T. rangeli* SC-58 [38]) was unexpected.

Motif analysis shows that the conserved amastin signature sequence of C-[IVLYF]-[TS]-[LFV]-[WF]-G-X-[KRQ]-X-[DENT]-C, which may be critical for amastin function [86], is present in all the species examined (Additional file 1: Table S11). Additionally, we found a motif, with consensus EAKKPAGSNEESPMSREALS, tandemly repeated 6 and 3 times respectively in two of the eight amastin genes analyzed from *T. rangeli* AM80. The function of this repeat is unknown, although we postulate that repeats may aid recombination and antigenic reshuffling associated with evasion of the host immune system [87]. Kinetoplastid Membrane Protein-11 (KMP-11) is encoded in *Leishmania*, *T. brucei* and *T. cruzi.* The observation that the KMP-11 genes are expanded in *T. rangeli* SC-58 [38] represented an unexpected result in this non-pathogenic strain. This finding is more unusual given that the gene is found in low numbers across other trypanosomatids [88, 89] and in our analysis we find just one copy in *T. conorhini* 025E, *T. rangeli* AM80 and *T. cruzi* CL, and none in *T. cruzi* G.

Finally, mucin, a family thought to confer immune system protection [61, 90], contains highly variable regions that make copy number estimation challenging. *T. rangeli* and *T. conorhini* appear to contain mostly mucin-like glycoproteins and little of the diversity of other mucin subgroups that is typical of *T. cruzi*, concurring with reports in other *T. rangeli* strains [91, 92]. The low gene copy numbers within this gene family in these two species are also consistent with previous genomic [38] and transcriptomic [72] data from *T. rangeli,* and likely contribute to their inability to invade mammalian cells [64]. Although likely underestimated here, we observe a larger gene family in *T. cruzi* CL compared to the other species, presumably contributing to the poorer immune system clearance of this strain.

### Pseudogenes

Pseudogenes are defined herein as genes bearing in-frame stop codons or frameshifts, as well as the absence of features required for gene calling based on a non-supervised training model, such as upstream functional sites, start codons, nucleotide and amino acid composition, and length to the first in-frame stop codon. The number of putative pseudogenes predicted in the *T. conorhini, T. rangeli*, *T. cruzi* G, and *T. cruzi* CL genome assemblies were 113, 434, 942 and 2376, respectively (Additional file 1: Tables S12–15). The latter equates to 18% of total gene predictions (gene calls per haploid genome plus pseudogenes) in the CL strain. The *T. cruzi* CL Brener genome was previously estimated to have 3590 pseudogenes, or ~ 16% of all its genes. Over 2000 of these were attributed to large multigene families [32]. We analyzed the NCBI nr annotated functions of our panels of predicted pseudogenes and found over 300 copies of putative pseudogenes from multigene families in *T. cruzi* CL. In both *T. cruzi* G and CL, the most frequent putative pseudogenes were of the trans-sialidase, RHS and MASP gene families, and hypothetical proteins.

The pseudogenes in these genomes may provide a repertoire of genetic information for producing variation, especially in multigene-families*. T. cruzi* was the first species in which a tandem array of pseudogenes, consisting of six mucin genes each with an in-frame stop codon, was discovered [93]. These were postulated to be selectively maintained in the genome, possibly to generate mucin gene diversity. A diversifying role has been suggested for the numerous pseudogenes of variable surface glycoproteins (VSGs) in *Trypanosoma equiperdum* and African trypanosomes that undergo rapid antigenic variation through gene recombination [94–97]. Additionally, TS gene and pseudogene organization, flanked by RHS genes at subtelomeric regions, in strain CL Brener is reminiscent of regions next to *T. brucei* VSG genes [98]. Pseudogenes could also play a role in post-transcriptional control of gene expression. Some pseudogenes transcribed in *T. brucei* have been proposed to participate in RNAi-based natural antisense suppression [99]. The genes responsible for RNAi machinery are absent in all strains of *T. cruzi* examined to date and may only be present as pseudogenes in *T. rangeli* SC-58, but are present and intact in both *T. rangeli* AM80 and *T. conorhini* 025E [100]. Analysis of the transcriptional activities and structural organization of these pseudogenes is beyond the scope of this study, but may clarify their roles in generation of protein diversity or post-transcriptional regulation.

### Heterozygosity

**Fig. 5:**
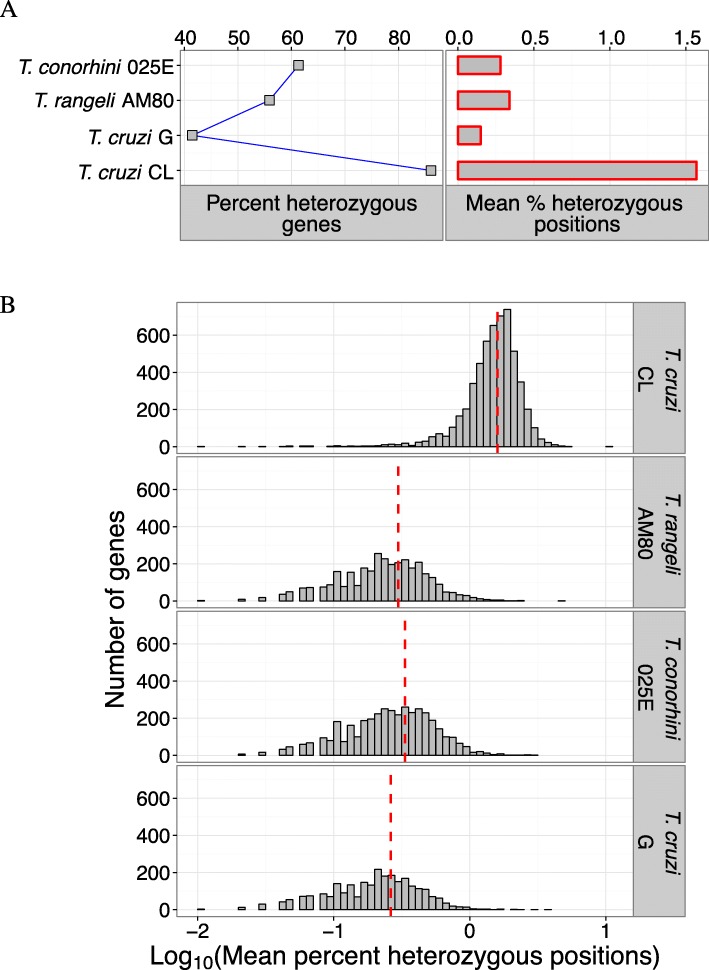
Heterozygosity of single copy orthologs. **(a)** Summary values from 6394 shared single copy ortholog genes. Percent heterozygous genes indicate percentage of genes with at least one heterozygous position, mean percent heterozygous positions were calculated by dividing the number of heterozygous sites by the total number of positions. (**b**) Histogram showing the distribution of heterozygosity values among heterozygous genes. Red vertical dashed lines represent the mean values

The four organisms described herein are thought to be primarily diploid, although some *T. cruzi* strains, e.g., CL and CL Brener, are hybrid strains in which ploidy is less well defined [58, 101, 102]. Thus, we examined levels of apparent heterozygosity in these strains using a set of 6394 conserved single copy orthologs, covering ~ 9 million sites in each genome. The number of heterozygous genes with at least one SNP varied from ~ 42% in *T. cruzi* G to ~ 88% in *T. cruzi* CL, whereas *T. conorhini* and *T. rangeli* have an intermediate 55–60% of apparently heterozygous genes. The average percent of heterozygous bases varied from ~ 0.1–0.3% in *T. rangeli*, *T. conorhini* and *T. cruzi* G to ~ 1.6% in *T. cruzi* CL (Fig. 5a and b). The low percentage of heterozygous positions for *T. conorhini*, *T. rangeli* and *T. cruzi* G are close to estimates of heterozygosity in *T. c. cruzi* Sylvio X10 (~ 0.22%) and *T. c. marinkellei* B7 (0.19%) [36], although those analyses were not restricted to single-copy orthologs, which may have positively biased their estimates. The high level of heterozygosity in *T. cruzi* CL is very likely mostly due to the fact that it is a hybrid in which a significant fraction of its genes are derived from two distantly related progenitors. The distributions of heterozygous genes falling into discrete mean levels of percent heterozygous positions were unimodal for all species (Fig. 5b), suggesting that the genes examined were not of biased origin. We found < 0.1% tri-alleles, and 0% tetra-alleles at the heterozygous sites of each organism, consistent with the genomes being largely diploid.

There was no significant enrichment of any KOG categories or enzyme E.C. numbers in genes with high or low heterozygosity values (data not shown). The overlap of highly heterozygous genes from the 6394 orthologs in each species was limited, and we found no evidence of synteny in heterozygosity patterns across contigs in any pairwise species comparison (data not shown). Together, these observations suggest that generation of heterozygosity in these organisms is largely a stochastic process.

*T. cruzi* displays strong linkage disequilibrium and features of a mainly clonal species [103]. However, the presence of natural *T. cruzi* hybrids such as those of TcV and TcVI and conservation of meiosis-related orthologs in the genomes suggest the capacity for sexual reproduction. Notably, the genome of *T. cruzi* G in particular exhibits overall low levels of heterozygosity. These levels do not seem to fit with a strictly clonal model of evolution, where diversity is expected to accumulate independently between alleles in an individual over time (the Meselson effect). Moreover, long-term clonality without mechanisms to attenuate the impact of high mutational load (Müller’s ratchet) would seem to be detrimental to these species.

A comprehensive analysis of heterozygosity in sequences spanning the genomes compared to expected heterozygosity is beyond the scope of the present study. However, our analysis of single copy orthologs identified an apparent mosaic pattern of heterozygosity across these genomes, especially in *T. cruzi* G and *T. rangeli* AM80, where continuous regions of homozygosity often exceeding 50 Kbp interspersed with heterozygous clusters were identified (Additional file 1: Table S16). Mosaic heterozygosity has been seen before in *Naegleria gruberi* [104], and clustering of heterozygosity has also been described in *T. c. marinkellei* [36]. Precise mechanisms that control heterozygosity in trypanosomes have yet to be elucidated. Many regions of low heterozygosity in all species have average coverage for single copy orthologs (Additional file 1: Table S16). Therefore, loss of heterozygosity via chromosome loss seems unlikely in these species, although this process cannot be ruled out. Mutational hotspots, mitotic recombination, mitotic gene conversion, and segmental duplication are also possible sources of differentially heterozygous regions of *T. cruzi* G and *T. rangeli* AM80 chromosomes.

Members of DTU TcVI, including hybrid strain *T. cruzi* CL, are reported to have a high degree of fixed heterozygosity but low intralineage diversity [105]. The higher heterozygosity originates at least in part from the distances between the Esmeraldo-like (TcII) and non-Esmeraldo-like (TcIII) alleles, provided by the ‘parental strains’ of DTU TcVI. As previously suggested [9], these hybrid genotypes were likely stabilized through long term asexual reproduction. Increased heterozygosity has been linked to hybrid vigor, which has been reported in *Leishmania* [106], and is consistent with an enhanced host range and the ability to invade cells, replicate, and cause pathogenicity.

Ratios of non-synonymous to synonymous SNPs within the set of single copy orthologs were vastly different across the organisms. Despite having a similar overall number of polymorphic sites, *T. rangeli* AM80 and *T. conorhini* 025E had non-synonymous to synonymous SNP ratios of 0.68:1 and 0.9:1 respectively. The lower rate of non-synonymous SNPs in *T. rangeli* AM80 is perhaps suggestive of greater purifying selection or functional constraint within proteins. The two *T. cruzi* strains also displayed differences, with a ratio of 0.95:1 for *T. cruzi* G and 0.8:1 for *T. cruzi* CL. This suggests that analysis of dN/dS ratios, especially for sites of selected gene groups within each species, would likely be an interesting next step to determine the extent and specificity of selective pressures.

### Repetitive elements

The genomes were analyzed for known trypanosome repeats, i.e., non-Long Terminal Repeat (non-LTR) elements, LTR elements, and satellites, using Repbase [107]. Repeat profiles (Additional file 1: Table S3 and S17–20) are similar for *T. conorhini* 025E and *T. rangeli* AM80. The most common satellite sequence in all species is SZ23_TC. Retroelements are markedly increased in the *T. cruzi* strains. Analysis of *T. conorhini* 025E and *T. rangeli* AM80 LTR- and non-LTR elements identified only 22 and 35 known trypanosomal elements, respectively. We hypothesize that these organisms may lack “copy and paste” type retrotransposons. To show that fewer repetitive element copies in the genomes of *T. rangeli* and *T. conorhini* was not just due to collapse of reads in highly similar repeats, we calculated the average coverage of the de novo repeat finder predictions in each repeat class. *T. cruzi* CL has coverage estimates close to the genomic average for every repeat class. *T. cruzi* G and *T. conorhini* 025E have around two-fold higher average coverage for the non-LTR and satellite sequences than the genomic average, and *T. rangeli* AM80 has two-fold higher coverage for just non-LTR sequences than the genomic average. The collapse of these highly similar repeats in the assembly may suggest mainly recent repeat expansion, although the numbers are still much lower than in *T. cruzi* CL. Retroelements may have wide-ranging implications on generation of genomic diversity, and their greater number in *T. cruzi* may have potentiated antigenic variation in this complex parasite [108]. It is interesting to speculate that the presence or absence of RNAi in these organisms may be connected to retroelement counts, as RNAi has been proposed to have a defensive role against transposable elements [109]. *T. conorhini* and *T. rangeli* have RNAi and possess fewer retroelements, whereas the *T. cruzi* strains lack RNAi and have a large repertoire of retroelements.

### Metabolic pathways analysis

We examined the metabolic capacity predicted by the genomes of these organisms (Additional file 1: Table S21). Below, we outline some of the more significant observations from these analyses.

#### Fatty acid metabolism

Trypanosomatids possess a unique set of elongase enzymes for de novo fatty acid synthesis [110]. *T. cruzi* amastigotes utilize lipid-dependent energy metabolism [75], but the functional importance of fatty acid oxidation in trypanosomatids is not fully understood. The organisms analyzed herein appear to be capable of synthesizing and oxidizing fatty acids (Additional file 1: Table S21). Glycerol dehydrogenase (EC 1.1.1.6), which is involved in converting glycerol to dihydroxyacetone, is present in *T. rangeli* and *T. conorhini*, but absent in the *T. cruzi* strains. This enzyme was reportedly acquired by lateral gene transfer in *Leishmania*, *Crithidia* and *Leptomonas* spp. [111], enabling the parasites to use glycerol as a carbon source.

#### Amino acid metabolism

In *T. cruzi*, amino acids are relevant in energy metabolism [112–115], host-cell invasion [116], stress resistance [117, 118], and differentiation [115, 119]. Proline, in particular, plays a fundamental role in these processes, including energy support during the parasite’s intracellular stages [115].

*T. cruzi*, unlike *T. brucei*, can metabolize D-proline using a putative proline racemase (PRAC). Although the *T. conorhini* genome bears a PRAC gene, as we have previously described [27] *T. rangeli* AM80, and strains of all other known *T. rangeli* lineages [27] contain only a pseudogene for this enzyme. Interestingly, genes for 5-oxoprolinase, which is involved in L-proline metabolism, are absent in *T. conorhini* 025E and *T. rangeli* AM80. Analysis of the available genomes of TriTrypDB [59] showed only intracellular-replicating species seem to possess this enzyme, which makes its apparent loss in these two species expected. L-proline metabolism via 5-oxoprolinase produces L-glutamate in the glutathione-mediated stress response pathway. *T. rangeli* AM80 also lacks enzymes that use oxygen as an acceptor (EC 1.4.3.-), which further limits the pathways available for glutamate synthesis. Absence of these enzymes may shed new light on the recent finding that *T. rangeli* SC-58 is particularly susceptible to oxidative stress [38]. However, there appear to be alternative pathways to produce glutamate and glutathione in both *T. rangeli* AM80 and *T. conorhini* 025E, e.g. glutamate dehydrogenase, which converts α-ketoglutarate to glutamate.

Consistent with previous reports in *T. cruzi* [120, 121] all of these species lack ornithine and arginine decarboxylase genes, indicating that they are unable to generate putrescine or other polyamines and must salvage them from their hosts. Primary-amine oxidase, which is significant for amino acid metabolism and alkaloid biosynthesis, is absent in *T. rangeli* AM80. The *T. rangeli* AM80 and *T. conorhini* 025E genomes encode branched-chain amino acid aminotransferase, which is required for synthesis and degradation of valine, leucine and isoleucine. That *T. cruzi* strains lack this gene [31] is interesting since leucine is reported to act as a negative regulator of proline-dependent metacylogenesis [122]. Additionally, the ability of this parasite to use the intact leucine skeleton, presumably obtained from the host [123], for isoprenoid and sterol formation would confer advantages in energy economy [124]. *T. cruzi* and *T. rangeli* can interconvert serine and glycine, a capacity not found in *T. brucei* [31]. *T. conorhini* 025E, like *T. brucei*, lacks the glycine hydroxymethyltransferase gene for conversion of glycine to L-serine and tetrahydrofolate or vice versa, although alternative routes exist in this organism for synthesis of these compounds.

#### Carbohydrate metabolism

Kinetoplastids compartmentalize a variety of enzymes involved in carbohydrate metabolism within organelles known as glycosomes [125]. Glucose is the predominant carbohydrate utilized by *T. cruzi* [126] and *T. brucei* [127], although *Leishmania* spp. and *Phytomonas* spp. have developed adaptations to metabolize plant-derived carbon sources [31, 128]. Genes for NADP-alcohol dehydrogenase (EC 1.1.1.2), which participates along with other enzymes in acetaldehyde to ethanol interconversion in glycolysis [129], are absent in *T. conorhini* and *T. rangeli*, but present in both *T. cruzi* strains. Several bacterial-type sugar kinases (glucokinase, galactokinase and L-ribulokinase), which contain targeting signals for import into glycosomes, are encoded in all four of the genomes of this study. However, genes for many other sugar metabolism enzymes, i.e. beta-glucosidase, fructuronate reductase, xylulokinase, and mannitol 2-dehydrogenase are only present in *T. conorhini* and *T. rangeli*. Proteins encoded by beta-glucosidase genes, for example, convert glucoside to α–D-glucose, and cellulose derivatives cellobiose and 1,4-β-D-Glucan to β –D-Glucose [129, 130]. These observations are consistent with the hypothesis that the latter two parasites are better adapted to environments more enriched in exogenous sugars and complex carbohydrates, an adaptation inconsistent with replication in the glucose-rich bloodstream. A nutritional role of plants in triatomines appears possible given demonstration of *Rhodnius* phytophagy [131]. Adaptation to vector diet may therefore have played a more important role in the evolution of these species than in *T. cruzi*, which is interesting given that the latter commonly co-infects the same triatomine vector as *T. rangeli* and also shares the same vector species as *T. conorhini*.

#### Overall metabolic potential

The metabolic potentials of *T. rangeli* and *T. conorhini* are more similar to each other than either is to *T. cruzi*. Each has around 20 differences in enzyme presence/absence compared to *T. cruzi*. All have complete pathways for glycolysis/gluconeogenesis, mannose metabolism, and pyruvate metabolism, although D-lactate dehydrogenase genes are absent in the *T. cruzi* strains. Glyoxylate and dicarboxylate metabolism appears deficient in all species, since isocitrate lyase and malate synthase, the two enzymes characteristic of the glyoxylate cycle, are absent. Interestingly, CAAX prenyl protease 1 (STE24 endopeptidase), presumably a membrane-associated protein [132] involved in terpenoid backbone synthesis, is present in the *T. cruzi* strains, but absent in *T. rangeli* AM80 (and also the *T. rangeli* SC-58 assembly of TriTrypDB v.24) and *T. conorhini* 025E. This gene is widely conserved in eukaryotes and highly diverged from CAAX prenyl protease 2, suggesting lack of redundancy. Terpenoids are precursors of steroids and sterols, possibly suggesting a role in host-parasite interaction [76]. Several genes common to *T. rangeli*, *T. conorhini* and the *T. cruzi* strains i.e. genes encoding galactokinase, glutamate dehydrogenase (NADP), serine acetyltransferase and l-ribulokinase and 2-aminoethylphosphonate-pyruvate aminotransaminase (AEP transaminase), have purportedly been passed to trypanosomes via horizontal gene transfer, and are absent in *T. brucei* [31]. Genes for aminoethylphosphonate (AEP) offer an alternative to ethanolamine phosphate for linkage of mucins to their GPI anchors [133]. Enzymes for the synthesis of AEP from phosphoenol pyruvate are conserved in all four genomes.

## Conclusions

Herein, we showed that genomes of *T. rangeli* AM80, *T. conorhini* 025E and *T. cruzi* strains G and CL, range from ~ 30–70 Mbp and contain between 10,000 and 13,000 genes. We characterized multigene families, the heterozygosity, and pseudogene content of these genomes, and used multi-gene strategies to explore their phylogenetic relationships. Our results show that *T. conorhini* and *T. rangeli* have less complex genomes, fewer genes, a decreased representation of multigene families, and fewer pseudogenes, than the *T. cruzi* strains. These observations generally are consistent with the simpler, non-intracellular lifestyles of these parasites. Genes and gene families, including amastin, MASP, and DGF-1, and others, are represented in these parasites in ways that support their association with pathogenicity, intracellular life cycle and host range. The metabolic potentials of these organisms provide clues as to the basis of these biological capabilities, with *T. rangeli* and *T. conorhini* bearing a greater number of enzymes for utilizing complex carbohydrates and glycerol as carbon sources, and displaying highly divergent amino acid metabolism to *T. cruzi*. Heterozygosity levels suggest less allelic diversity in *T. rangeli* AM80, *T. conorhini* 025E and *T. cruzi* G, than in *T. cruzi* CL. Phylogenetic distance in substitutions per site between the *T. rangeli* strains SC-58 and AM80 is about the same as *T. cruzi* strains to *T. c. marinkellei*, and the distance of *T. rangeli* AM80 to the *T. cruzi* strains is just over twice the distance between *T. rangeli* AM80 and *T. conorhini* 025E.

## Methods

### Parasites and culture

Parasites were obtained from the Trypanosomatid Culture Collection (TCC) at the University of Sao Paulo, the American Type Culture Collection (ATCC), and Nobuko Yoshida (Universidade Federal de São Paulo) as shown (see Additional file 1: Table S1). Parasites were cultured, and DNA was isolated and sequenced essentially as previously described [134]. Briefly, epimastigote form parasites were cultured at 28 °C in liver-infusion tryptose (LIT) medium, supplemented with 20% fetal bovine serum (FBS) with 20 μg/ml hemin for *T. rangeli* AM80, and 10% fetal bovine serum (FBS) with 10 μg/ml hemin for all other species, and harvested in log phase at ~ 1 X 10^7^/ ml. Total DNA was isolated, and depleted of kinetoplast DNA (kDNA), by gel electrophoresis as previously described [134].

### Genome sequencing and assembly

The purified DNA was used to prepare shotgun and 3 Kbp mate pair libraries (8 Kbp mate pair libraries in the case of *T. cruzi* CL) for sequencing on the Roche 454 GS FLX+ platform as indicated by the manufacturer. Reads aligning with a minimum of 50% identity and over 50% length to kDNA from TriTrypDB were removed, and only those reads with at least 70% bases with a PHRED quality score greater than 25 and a minimum read length of 40 bp were kept using NGS QC toolkit [135] version 2.3. This yielded ~ 3–5 million reads for each organism, with average read lengths of 330–360 bp (Additional file 1: Table S3). Assembly was performed using the Newbler version 2.9 assembler (Roche, Inc.), which limits the size of scaffolds to a minimum of 2 Kbp. Hence, all contigs larger than 500 bp that were not part of any scaffold were appended to the scaffolded assemblies for completeness. The highly repetitive and heterozygous *T. cruzi* CL genome was assembled using the *T. cruzi* CL Brener assembly from TriTrypDB v.24 as a reference. Reads were mapped to the entire *T. cruzi* CL Brener genome with BWA v.0.7.12 [136], and reads aligning to each haplotype were extracted for use in individual haplotype assembly runs. Contigs less than 500 bp in length were removed from the final assemblies. Reads were realigned to the final assemblies using BWA [136] and the average genome-wide coverage was calculated to range between 14-50X, depending on the strain (Additional file 1: Table S3). The in-house tool Genome assembly Completion and Integrity Analyzer (GenoCIA) was used to estimate assembly completion and gene calling integrity. This tool performs two tasks: (i) randomly selects 2, 4, 6, 8, 10, 20, 30, 40, 50, 60, 70, 80, 90 and 99% of the reads and performs assemblies using Newbler with these read subsets; and (ii) uses tBLASTn to determine the presence of a curated set of 2217 kinetoplastid orthologous single copy genes at 25, 50, 75, 90 and 99% alignment lengths (merging reference gene alignment lengths over multiple contigs or genes where necessary). BLASTn was used to further characterize whether these genes were complete or fragmented on contigs or gene calls. The general characteristics of these genomes were determined using an in house Genome Annotation Pipeline (GAP). Briefly, genes were called using GeneMarkS v.4.7b [137]; tRNAscan-SE [138] v.1.23 was used to detect tRNAs; and 5S/18S/28S sequences were detected using RNAmmer [139] v.1.2. SignalP [140] v.4.1 (default settings) identified signal peptides and anchors in called genes. TMHMM [141] v2.0 (default settings) determined genes with at least one transmembrane domain. KOHGPI v.1.5 of GPI-SOM [142] was employed with the default training set and settings to predict genes with GPI anchors. BLAST [143] searches against Pfam [144], KOG [145], TriTrypDB and NCBI’s nr databases were performed to determine validity and integrity of the gene calls, and ascertain probable gene functions and inferred annotations. Collapse of repetitive sequences in the assemblies was assessed from the Newbler assembler coverage histogram, within the Newbler output file 454NewblerMetrics.txt.

### Molecular karyotypes

Genomic DNA isolation and pulsed field gel electrophoresis (PFGE) were performed essentially as previously described [55]. See Fig. 1 for run conditions. Band sizing based on standard curves of marker chromosome migration and densitometry for each gel was performed using GelAnalyzer v. 2010a [146], with rolling ball background subtraction. Briefly, we obtained the “volume” of each presumed single diploid chromosomal band by multiplying pixel area by the sum of the pixel intensities within the boundary assigned to the band, and then adjusted for background pixel intensity to get “adjusted volume.” A standard curve of adjusted volume vs. size using marker chromosomes was used for reading off the expected volume of each observed band of a specific size. This was then compared to the actual volume to get diploid chromosome copy number, assuming a linear correlation between copy number and volume at a specific band size.

### 18S rRNA copy number via qPCR

qPCR and the relative threshold algorithm on ViiA 7 (Thermo Fisher Scientific) were employed using MGB primers and probes specific for conserved regions of the18S gene in each species where the highest number of reads mapped (Additional file 1: Table S22), and DHFR as a single copy reference gene for normalization. Estimations were taken at two different dilutions of gDNA sample, each in triplicate, with three biological replicates performed in separate runs. Wells with no DNA served as no template controls, and standard curves indicated equivalency of primer/probe set efficiencies.

### Sequence identity and phylogenetic analysis

Annotated proteins from *T. brucei* TREU 927, *T. brucei gambiense* DAL 972, *T. grayi* ANR4, *T. evansi* STIB805, *T. rangeli* SC-58, *T. cruzi marinkellei* B7, *T. cruzi* Sylvio-X10, *T. cruzi* Dm28c, *Leishmania mexicana* MHOM/GT/2001/U1103, *Leishmania major* Friedlin*, T. congolense* IL3000 and *T. vivax* Y486 were downloaded from TriTrypDB v.24. OrthoFinder v.0.7.1 [147] processing (default parameters) using the data from TriTrypDB and the gene calls from our sequenced genomes, identified 224 annotated single copy orthologs that are present in all species. Clustalo v.1.2 [148] alignments with Gblocks v0.91b [149] editing (parameters: b4 = 5, b5 = h) of these genes in 10 selected species were checked to ensure no alignment had > 50% of positions filtered out or had a length of < 100 amino acids. EMBOSS infoalign [150] and a custom Perl script were used to remove any edited alignments that contained a sequence > 25% shorter than the median alignment length to avoid including partial or broken genes. Thirty-seven orthologs were removed in this analysis. Visual inspection of the remaining 187 edited alignments identified 48 that contained at least one poorly aligning sequence and were therefore removed, leaving a final set of 139 orthologous genes present in all 10 organisms. These gene alignments were concatenated using FASconCAT v1.0 [151], and the resulting supermatrices were used for phylogenetic reconstruction. ProtTest v.3.4 [152] Bayesian Information Criterion (BIC) determined that 88% of these proteins best fit the JTT substitution model, and 85% of proteins had gamma as the best model for rate heterogeneity. RAxML v.8.1.17 [153] PROTGAMMAJTT, which applies a gamma distribution with four discrete rate categories allowing for different rates of evolution at different sites, was used for building 200 maximum likelihood (ML) trees on distinct randomized stepwise addition parsimony starting trees to obtain the tree with the best likelihood. Support values for the tree were then obtained by rapid bootstrap analysis with 1000 replicates. Bootstrap values were then used to draw bipartitions on the best ML tree. TreeGraph 2.4.0 [154] and Inkscape 0.91 [155] were used for tree visualization and editing, with mid-point rooting on *T. brucei*. The 139 amino acid alignments without Gblocks editing were used by PAL2NAL v.14 [156] to obtain corresponding codon alignments. These were both concatenated with FASconCAT v1.0 and used for average pairwise percent identity calculation with a custom Python script incorporating the AlignIO utility of Biopython [157] .

### Multigene family and 18S in silico analysis

Multigene family copy number: We selected 13 multigene families for analysis based on gene cluster diversity analyses of this study and literature searches. Called genes by GeneMarkS v.4.7b [137] were grouped into multigene families based on choosing the best non-hypothetical protein annotation out of the top 10 E-value hits to NCBI’s non-redundant protein database via BLASTp (E-value threshold 1e-5). Gene coordinates were then converted to GFF format for reads mapping with BWA v.0.7.12 [136] (default parameters). Copy number for each multigene family was calculated based on read depth using SAMtools v1.2 [158]. Average per base coverage was calculated then divided by average coverage of a set of 6394 single copy orthologs (same gene set as for heterozygosity analysis). A representative complete gene length for each multigene family was selected based on the consensus longest trypanosome gene length for each multigene family from UniProtKB full-length genes (as described in Additional file 1: Table S10), and used to correct copy number estimates for fragmented genes. Fragmentation of genes is a common problem in copy number estimation of complex and incomplete genomes [159], and given that portions of genes in the genome may not assemble our estimates are likely conservative. As a validation for our read-based approach we obtained values of 1 for dihydrofolate reductase*,* poly (A) polymerase and DNA topoisomerase type IB, which are widely considered to be single copy genes in trypanosomatids, using the same methodology.

Amastin motif analysis: motif prediction was performed on translated gene sequences using MEME [160] v.4.10.0 with the anr option and a maximum width of 20.

18S copy number: estimated as described above for multigene family analysis, except that gene coordinates were predicted by RNAmmer v1.2 [139] and aligned bases were only calculated at positions of q-score over 25 with a minimum two-fold coverage.

### Pseudogenes

Longest nr database hits for each genomic coordinate were predicted by gapped BLAST with the program lastal [161] v.744 (parameters -F15, −l5 –K20, −X 150 –P0), followed by selection of hits containing frameshifts or premature stop codons. Coordinates were converted to GFF format, removing any overlapping genes called by GeneMarkS v.4.7b [137] using BEDTools v.2.19.1 intersect. As described above, since over 98% of 2217 single copy orthologs shared between *T. brucei*, *T. vivax*, *T. congolense*, *T. dionisii*, *T. cruzi* and *Leishmania* species were present, likelihood of finding false positives due to uncalled genes was low.

### Heterozygosity

High quality sequence reads, i.e., reads with 70% of the bases with quality score ≥ 25, were aligned to each genome assembly. Polymorphic positions were then quantified in each of 6394 single copy OrthoFinder v.0.7.1-generated orthologs present in each of the four genomes examined, using SAMtools v1.2 [158] to generate and index bam files, FreeBayes v1.0.1 [162] to detect variants (parameters --ploidy 2 --vcf) and VCFtools v.0.1.9 [163] to summarize SNP results (parameters --remove-indels --recode-INFO-all). Synteny of the distribution of heterozygosity values at local areas of the genomes was assessed by Spearman’s Rank followed by adjusting the *p*-values using the Bonferroni correction using Rstudio. Windows of distance (bp) and number of genes had to be similar for pairwise species comparisons. To assess the percentage of bi- tri- and tetra-allelic sites the FreeBayes VCF output was subjected to alternative allele counts (e.g. --min-alleles 3 --max-alleles 3 for tri-alleles). SnpEff v4.3T [164] was used to assess synonymous to non-synonymous changes at SNP sites (default parameters).

### Repetitive elements

Repeat counts in intergenic regions of the assemblies were identified by performing a Cross Match v. 0990329 search and categorization with RepeatMasker [165] v. 4.0.6. RepeatMasker library sequences of *Trypanosoma* species derived from Repbase (20150807 download) were used as a database for the search. De novo repeats were predicted using RepeatMasker with a library built using RepeatModeler [166] v1.0.8. The latter identified and modeled de novo repeat families from the four genomes using RECON v.1.08, RepeatScout v.1.0.5 and Tandem Repeat Finder v.4.0.4 [167–169], with an RMBLASTn [166] v.1.2 search of Repbase.

### Metabolic pathways analysis

Database reference genes from UniRef100 [170] and the Kyoto Encyclopedia of Genes and Genomes KEGG [129] were located on assembly contigs and mapped to metabolic pathways using ASGARD [171]. Enzymes found to be differentially present among the four species were subjected to an additional tBLASTn analysis of the sequencing reads, requiring > 60% of the reference gene sequence to be covered by at least four reads with an E-value <1e-5 to indicate presence.

## Additional files

Additional file 1: **Table S1.** Strain information. **Table S2.** Densitometry of PFGE. **Table S3.** Sequencing and genome statistics summary. **Tables S4-S7.** Annotation of genes. **Table S8.** Orthologous genes used for phylogenetic reconstruction. **Table S9.** Percent identity matrix. **Table S10.** Multigene family copy number analysis and full gene lengths from UniProtKB. **Table S11.** Amastin motif analysis. **Tables S12-S15.** Putative pseudogenes for each species. **Table S16.** Orthologous gene heterozygosity values. **Tables S17–20.** Coordinates of repetitive elements. **Table S21.** Metabolic analysis. **Table S22.** SSU rRNA qPCR primers and probes. (XLSX 18721 kb)

Additional file 2: **Figure S1.** Genome assembly Completion and Integrity Analysis (GenoCIA). (A) Genome assemblies are comprehensive. Sequential assemblies were performed from 2, 4, 6, 8, 10, 20, 30, 40, 50, 60, 70, 80, 90, and 100% of the sequence reads generated for each of the species, and the percent of 2217 single copy orthologs shared between *T. brucei*, *T. vivax*, *T. congolense*, *T. dionisii*, *T. cruzi* and *Leishmania* species found in the assemblies was determined. (i) shows the percent of the orthologs that have a hit with 50% alignment length, (ii) shows the percent that have a hit with 90% alignment length. (B) Integrity of the gene calls. The genes called with GeneMark for each of the genomes analyzed herein were queried with the set of 2217 single copy orthologs, and the percent of orthologs that align at any length (at least), 25%, 50%, 90% or 99% length of the query gene/protein is shown. **Figure S2.** Distribution of the number of genes per OrthoFinder cluster. Percentage of clusters containing discrete gene counts, grouped by organism. The colour gradient and percentages over bars indicate the percent of clusters in each size bin that contain at least one gene with a TMHMM, KOHGPI or SignalP designation as surface-located or secreted. (PPTX 99 kb)

## Abbreviations

DGF: Dispersed gene family
DHFR: Dihydrofolate reductase
DTU: Discrete typing unit
GPI: Glycophosphatidylinositol
KMP: Kinetoplast membrane protein
KOG: Clusters of orthologous groups for eukaryotes
LINE: Non-long terminal repeat retrotransposon
MASP: Mucin-associated surface protein
PFGE: Pulsed field gel electrophoresis
RHS: Retrotransposon hot spot
RNAi: RNA interference
SNP: Single nucleotide polymorphism
STXBP: Syntaxin-binding protein
TS: Trans-sialidase
VSG: Variant surface glycoprotein
