# Supplementary figures and images for: Genomic comparison of Trypanosoma conorhini and Trypanosoma rangeli to Trypanosoma cruzi strains of high and low virulence
Source: BMC Genomics. 2018 Oct 24;19:770. doi: 10.1186/s12864-018-5112-0 (PMC6201504; doi:10.1186/s12864-018-5112-0)

## Slide 1
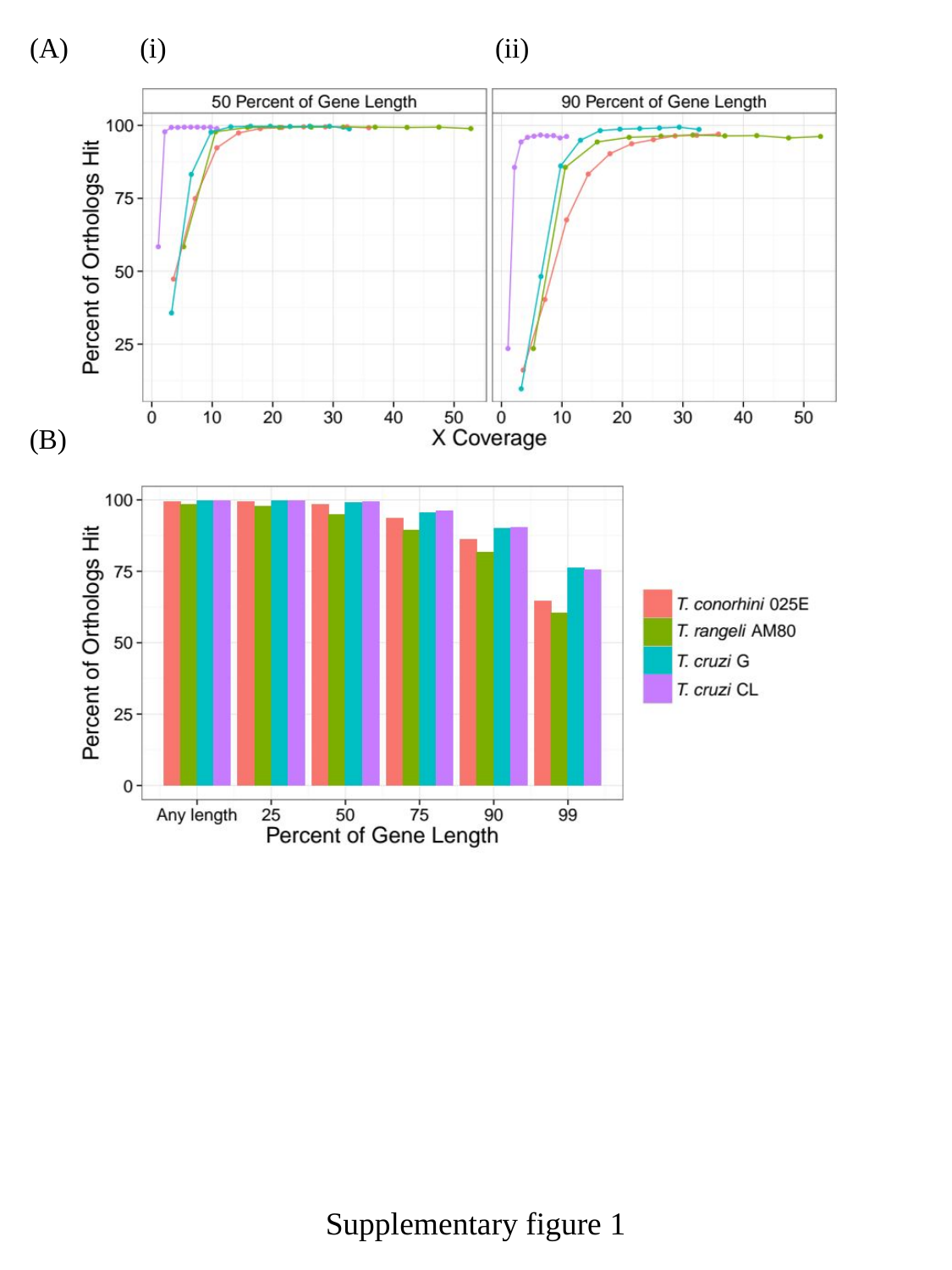

(A)
(i)
(ii)
(B)
Supplementary figure 1

## Slide 2
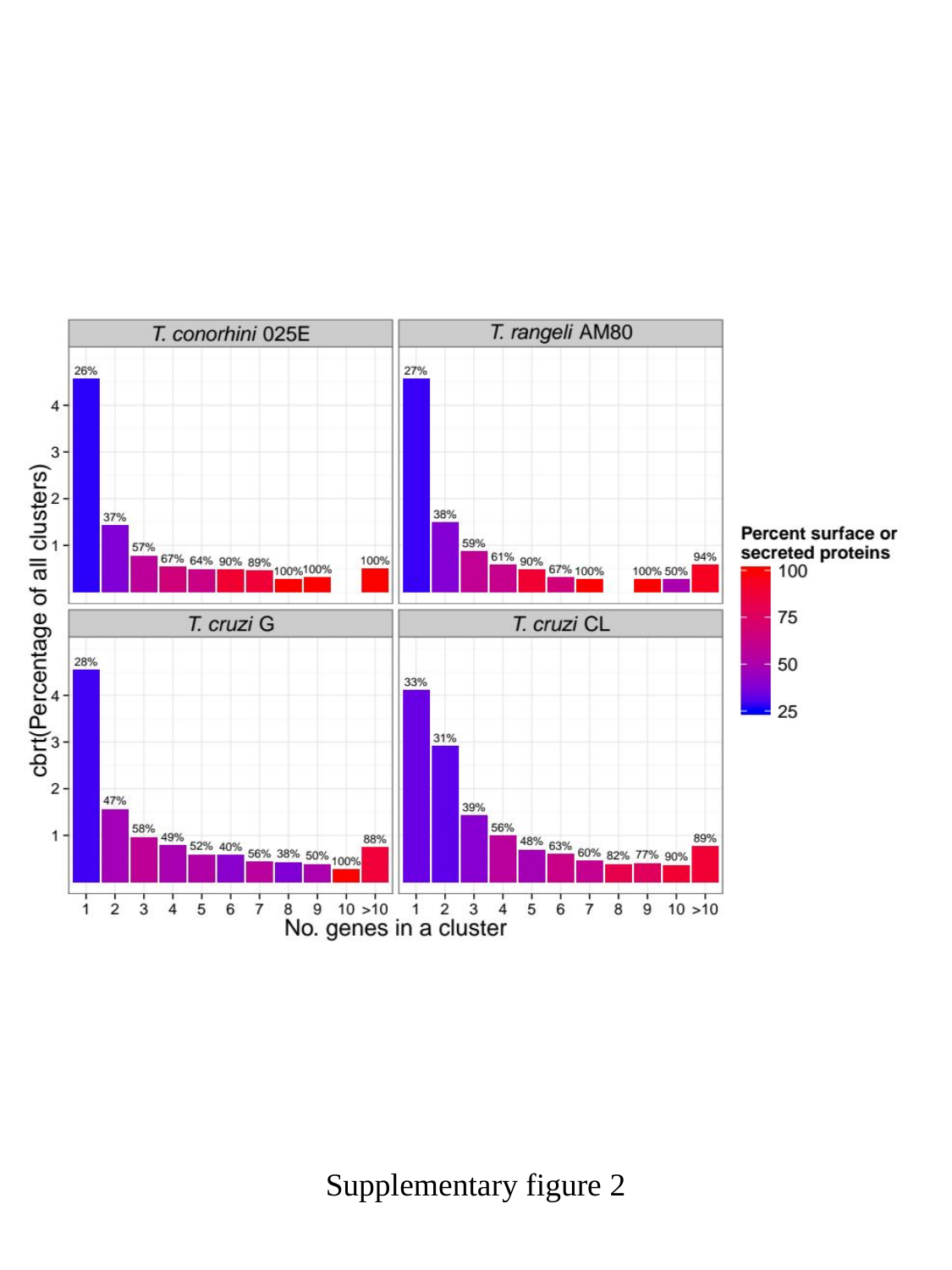

Supplementary figure 2

Supplement: Supplementary file 2 — Figure S1. Genome assembly Completion and Integrity Analysis (GenoCIA). (A) Genome assemblies are comprehensive. Sequential assemblies were performed from 2, 4, 6, 8, 10, 20, 30, 40, 50, 60, 70, 80, 90, and 100% of the sequence reads generated for each of the species, and the percent of 2217 single copy orthologs shared between T. brucei, T. vivax, T. congolense, T. dionisii, T. cruzi and Leishmania species found in the assemblies was determined. (i) shows the percent of the orthologs that have a hit with 50% alignment length, (ii) shows the percent that have a hit with 90% alignment length. (B) Integrity of the gene calls. The genes called with GeneMark for each of the genomes analyzed herein were queried with the set of 2217 single copy orthologs, and the percent of orthologs that align at any length (at least), 25%, 50%, 90% or 99% length of the query gene/protein is shown. Figure S2. Distribution of the number of genes per OrthoFinder cluster. Percentage of clusters containing discrete gene counts, grouped by organism. The colour gradient and percentages over bars indicate the percent of clusters in each size bin that contain at least one gene with a TMHMM, KOHGPI or SignalP designation as surface-located or secreted. (PPTX 99 kb) [file 12864_2018_5112_MOESM2_ESM.pptx]
